# Comparison of survival between malignant neuroendocrine tumours of midgut and pancreatic origin

**DOI:** 10.1038/sj.bjc.6690494

**Published:** 1999-06

**Authors:** V Johanson, L E Tisell, L Olbe, B Wängberg, O Nilsson, H Ahlman

**Affiliations:** Departments of Surgery and Pathology, Sahlgrenska University Hospital, Göteborg University, Göteborg, SE-413 45, Sweden

**Keywords:** midgut carcinoid, malignant endocrine pancreatic tumour, survival, staging

## Abstract

The survival of 64 consecutive patients with disseminated midgut carcinoid tumours was compared in a retrospective study with that of 25 consecutive patients with sporadic malignant endocrine pancreatic tumours treated according to similar surgical principles. The presence of hepatic metastases implied a worse prognosis in neuroendocrine tumours of pancreatic rather than midgut origin. This infers that these tumour types must be separated when treatments are evaluated. © 1999 Cancer Research Campaign


*British Journal of Cancer* (1999) **80**(8), 1259–1261
© 1999 Cancer Research Campaign
Article no. bjoc.1999.0494

### Comparison of survival between malignant neuroendocrine tumours of midgut and pancreatic origin

**V Johanson[1], LE Tisell[1], L Olbe[1], B Wängberg[1], O Nilsson[2] and H Ahlman[1]**

Departments of [1]Surgery and [2]Pathology, Sahlgrenska University Hospital, Göteborg University, SE-413 45 Göteborg, Sweden

**Summary** The survival of 64 consecutive patients with disseminated midgut carcinoid tumours was compared in a retrospective study with that of 25 consecutive patients with sporadic malignant endocrine pancreatic tumours treated according to similar surgical principles. The presence of hepatic metastases implied a worse prognosis in neuroendocrine tumours of pancreatic rather than midgut origin. This infers that these tumour types must be separated when treatments are evaluated.

**Keywords:** midgut carcinoid; malignant endocrine pancreatic tumour; survival; staging

Neuroendocrine tumours of the gastrointestinal tract are rare diseases and with the presence of metastases, patients may suffer from disabling symptoms of hormone overproduction. Potentially curative surgery can be performed in patients with localized disease, and sometimes in patients with liver metastases. However, long-term follow-up of the latter patients mostly shows late tumour recurrences (Norton, 1994). Debulking (cytoreductive) surgery can be considered when most of the tumour burden can be excised safely to minimize hormonal symptoms and to facilitate medical therapy (McEntee et al, 1990; Carty et al, 1992; Que et al, 1995). An important question is whether the aggressive treatment of metastatic disease prolongs survival. No randomized studies are available today. Such studies are complicated by the lack of strict surgical treatment protocols running over long periods, the relatively low number of patients and the absence of a TNM (tumour, node, metastases) classification of these tumour types. Many clinical studies, in which debulking surgery and resection rates are discussed, are composed of patients with carcinoids and those with endocrine pancreatic tumours (EPT). However, these two disease entities may include tumours with markedly different biology.

In our consecutive series of patients with advanced midgut carcinoids (all with locoregional and hepatic metastases) the estimated overall 5-year survival was 69% after active interventional treatment. The 5-year survival of carcinoid patients with bilobar liver metastases was 63% (cf. Wängberg et al, 1996). These figures are superior to the ones obtained after more conservative treatment. In a consecutive series of 42 patients with metastatic EPT from National Institutes of Health, the 5-year survival of those 17 patients resected for cure was 79% versus 28% in the non-resected group (Carty et al, 1992). Similar results were recently reported from the Mayo Clinic in 74 patients with hepatic metastases from either EPT or carcinoid tumours. The 4-year survival was 85% for one-third of the patients selected for resection versus 65% for the two-thirds non-resected (Que et al, 1995). In a recent French series of 34 patients with neuroendocrine tumours (carcinoids and EPT) and hepatic metastases, the liver resection rate was as high as 50%. Twelve of these 17 patients were operated upon with curative intent (Dousset et al, 1996).

The aim of this retrospective study was to compare survival in our consecutive series of patients with malignant EPT with that of patients with advanced midgut carcinoids when subjected to similar treatment principles. Furthermore, EPT were staged according to the TNM classification of pancreatic carcinoma. By doing so, stage IV tumours should be compared with advanced carcinoid tumours with liver metastases.

### MATERIALS AND METHODS

### Patients and treatment

The 64 consecutive patients with advanced midgut carcinoids were treated during 1987–1997 (cf. Wängberg et al, 1996). All patients underwent primary surgery, removal of the primary tumour and regional lymph node metastases, and excision of retroperitoneal tumours when present. Fourteen patients with unilobar hepatic lesions had a second operation (liver resection) with curative intent. Forty patients with multilobar liver metastases underwent ischaemic tumour reduction by hepatic arterial embolization, followed by octreotide treatment. Ten patients underwent only primary surgery, followed by octreotide in combination with interferon since further interventional treatment was contraindicated (high age, synchronous malignancies, thromboembolic disease, cirrhosis or psychiatric disorders). At tumour progression, additional treatment with cytotoxic drugs was given (streptozotocin and 5-fluorouracil alternated with doxorubicin; cf. Moertel et al, 1994).

During the same period, 25 consecutive patients with sporadic malignant EPT (mean age 50 years, range 17–77) were treated at our unit. All patients were surgically explored and underwent radical surgery or surgical debulking, if more than 90% of the intrahepatic metastases could be removed safely (cf. Foster and Lundy, 1981). Tumour staging was done according to the 1997 TNM classification of pancreatic carcinoma (Table 1). The series of malignant sporadic EPT included two glucagonomas (stage I and III), three insulinomas (stage I, $n$ = 1; stage IV, $n$ = 2), five

*Received 24June 1998*
*Revised 8 December 1998*
*Accepted 21 January 1999*

*Correspondence to:* V Johanson

**Table 1** TNM classification of pancreatic carcinoma

| | | | | |
|---|---|---|---|---|
| T1 | Limited to pancreas <2 cm | | | |
| T2 | Limited to pancreas >2 cm | | | |
| T3 | Duodenum, bile duct, peripancreatic tissues | | | |
| T4 | Stomach, spleen, colon, large vessels | | | |
| N1 | Regional lymph node metastasis | | | |
| M1 | Distant metastasis | | | |
| Stage I | T1–T2 | N0 | M0 | $n = 5$ |
| Stage II | T3 | N0 | M0 | $n = 0$ |
| Stage III | T1-T3 | N1 | M0 | $n = 6$ |
| Stage IV A | T4 | Any N | M0 | $n = 0$ |
| Stage IV B | Any T | Any N | M1 | $n = 14$ (6 NEC) |

$n$ = number of patients with EPT; NEC = neuroendocrine carcinoma.

gastrinomas (stage III, $n = 2$; stage IV, $n = 3$), six neuroendocrine carcinomas (NEC), (cf. Gould et al, 1984) (all stage IV) and nine non-functional islet cell tumours (stage I, $n = 3$; stage III, $n = 3$; stage IV, $n = 3$). Five patients with sporadic EPT stage I were classified as malignant due to histopathological criteria ($n = 4$), or later appearance of metastases ($n = 1$).

Eleven out of the 25 EPT patients were resected for cure. Seventeen patients had their primary tumour removed by enucleation ($n = 1$), pancreatic resection ($n = 13$) or total/subtotal pancreatectomy with regional lymph node excision ($n = 3$). It is notable that all 14 patients with hepatic disease (stage IV) presented with multilobar metastases. Hepatic debulking procedures were carried out in three patients. Seven out of 14 patients with liver metastases were treated with hepatic arterial embolization. All patients with liver metastases were treated medically (octreotide combined with interferon followed by cytotoxic drugs at tumour progression; cf. Moertel et al, 1994).

### RESULTS

The survival function related to tumour disease was estimated according to the Kaplan–Meier method. Patients with carcinoid tumours and intentionally curative liver resection had an estimated 5-year survival of 100% versus 63% in patients treated with hepatic artery embolization. In the group of ten patients with solely medical treatment after primary surgery, only one patient was alive after 5 years.

Eleven patients with resectable sporadic EPT tumours (stage I and III) had an estimated 5-year survival of 100% versus 0% for the 14 patients with liver metastases (stage IV) (Figure 1). Eight patients with stage IV disease (four NEC cases) had very advanced tumours and were not amenable to surgical treatment. The other six patients with stage IV disease (two NEC cases) had their primary tumours resected prior to further therapy. In the latter group, hepatic tumour debulking could be performed in three patients. For the EPT patients of stage IV the estimated 2-year survival was 53%, but declined rapidly thereafter. The NEC patients had an even worse survival with a median survival of 3 months (range 1–37 months).

### DISCUSSION

Very active surgery has become accepted as the treatment of choice for neuroendocrine tumours, many of which have a relatively slow growth (Galland and Blumgart, 1986; McEntee et al, 1990; Norton, 1994; Dousset et al, 1996). The active treatment of patients with advanced midgut carcinoids, including ischaemic tumour reduction of liver metastases, has resulted in long-term palliation of hormonal symptoms and high 5-year survival rates (Wängberg et al, 1996). Our results from the same period of a consecutive series of patients with advanced sporadic malignant EPT do not compare well with recent selected series presented from large referral centres. However, our 5-year survival figures for stage IV EPT disease closely resemble the results after liver transplantation for metastatic neuroendocrine tumours (bilobar disease) showing much worse prognosis for EPT than for carcinoids (Lang et al, 1997; Le Treut et al, 1997). In order to make reliable comparative studies it is important to have a relevant tumour staging system, and possibly also information on the biological behaviour of the tumour disease.

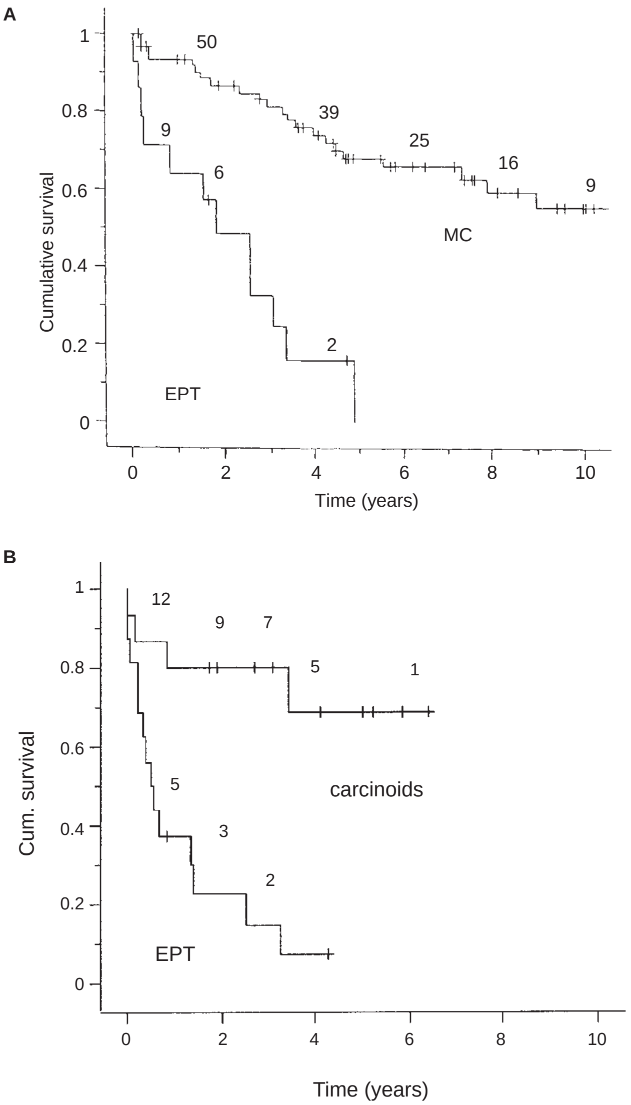

**Figure 1** Kaplan-Meier analyses of patients with neuroendocrine tumours and liver metastases. Number of patients at risk is indicated on top of curves. (**A**) Post-operative survival of patients with midgut carcinoids (MC, $n = 64$) and endocrine pancreatic tumours (EPT, $n = 14$). (**B**) Survival after orthotopic liver transplantation for metastatic neuroendocrine tumours as presented by Le Treut et al (1997) *Ann Surg* **225**: 355–364 (Reprinted with permission of the publisher). Different types of carcinoid forms ($n = 15$) versus EPT ($n = 16$)

*© 1999 Cancer Research Campaign*

Some investigators have proposed to divide patients into stable or progressive disease after an initial observation period prior to initiation of medical therapy (Arnold et al, 1996). In the present series, with a large proportion of patients with NEC tumours, tumour stage IV was a very strong predictor of poor survival.

The TNM classification of pancreatic carcinoma may not readily be applicable for EPT disease, since these tumours have a different growth pattern, e.g. no stage II tumours were found. In this material no EPT patient presented with unilobar liver metastases, while this occurred in 14 of 64 carcinoid patients. From the present study we conclude that high-stage sporadic EPT has a markedly worse prognosis than midgut carcinoids of similar stage, when the same treatment principles were followed. This means that the two diseases should be separately analysed when treatment is evaluated. It is important to identify patients with NEC tumours, since they carry a particularly poor prognosis and are seldom candidates for surgical treatment.

### ACKNOWLEDGEMENTS

This study was supported by The Swedish Medical Research Council (5220), The Swedish Cancer Society (3911), IB & A Lundberg Research Foundation, S. Ehrenström Foundation and the Landmann Foundation.

### REFERENCES

Arnold R, Trautman ME, Creutzfeldt W, Benning R, Benning M, Neuhaus C, Jürgensen R, Stein K, Schäfer H, Bruns C and Dennler HJ (1996) Somatostatin analogue octreotide and inhibition of tumour growth in metastatic endocrine gastroenteropancreatic tumours. *Gut* **38**: 430–438

Carty SE, Jensen RT and Norton JA (1992) Prospective study of aggressive resection of metastatic pancreatic endocrine tumours. *Surgery* **112**: 1024–1032

Dousset B, Saint-Marc O, Pitre J, Soubrane O, Houssin D and Chapuis Y (1996) Metastastic endocrine tumors: medical treatment, surgical resection, or liver transplantation. *World J Surg* **20**: 908–915

Foster JH and Lundy J (1981) Liver metastases. *Curr Probl Surg* **18**: 157–202

Galland RB and Blumgart LH (1986) Carcinoid syndrome. Surgical management. *Br J Hosp Med* **35**: 166–170

Gould VE, Jao W, Chejfec G, Banner BF and Bonomi P (1984) An overview: neuroendocrine carcinomas of the gastrointestinal tract. *Sem Diagn Pathol* **1**: 13–18

Lang H, Oldhafer KJ, Weimann A, Schlitt HJ, Scheumann GFW, Flemming P, Ringe B and Pichlmayr R (1997) Liver transplantation for metastatic neuroendocrine tumors. *Ann Surg* **225**: 347–354

Le Treut YP, Delpero JR, Dousset B, Cherqui D, Segol P, Mantion G, Hannoun L, Benhamou G, Launois B, Boillot O, Domergue J and Bismuth H (1997) Results of liver transplantation in the treatment of metastatic tumors: a 31-case French multicentric report. *Ann Surg* **225**: 355–364

McEntee GP, Nagorney DM, Kvols LK, Moertel CG and Grant CS (1990) Cytoreductive hepatic surgery for neuroendocrine tumours. *Surgery* **108**: 1091–1096

Moertel CG, Johnson MC, McKusik MA, Martin JK Jr, Nagorney DM, Kvols LK, Rubin J and Kunselman S (1994) The management of patients with advanced carcinoid tumors and islet cell carcinoma. *Am Int Med* **120**: 302–309

Norton JA (1997) Surgical management of carcinoid tumours: Role of debulking and surgery for patients with advanced disease. *Digestion* **55** (Suppl 3): 98–103

Que FG, Nagorney DM, Batts KP, Linzl J and Kvols LK (1995) Hepatic resection for metastatic neuroendocrine carcinomas. *Am J Surg* **169**: 36–43

Wängberg B, Westberg G, Tylén U, Tisell LE, Jansson S, Nilsson O, Johanson V, Scherstén T and Ahlman H (1996) Survival of patients with disseminated midgut carcinoid tumours after aggressive tumour reduction. *World J Surg* **20**: 892–899

© 1999 Cancer Research Campaign 
